# The burden of injury in Central, Eastern, and Western European sub-region: a systematic analysis from the Global Burden of Disease 2019 Study

**DOI:** 10.1186/s13690-022-00891-6

**Published:** 2022-05-20

**Authors:** Juanita A. Haagsma, Periklis Charalampous, Filippo Ariani, Anne Gallay, Kim Moesgaard Iburg, Evangelia Nena, Che Henry Ngwa, Alexander Rommel, Ausra Zelviene, Kedir Hussein Abegaz, Hanadi Al Hamad, Luciana Albano, Catalina Liliana Andrei, Tudorel Andrei, Ippazio Cosimo Antonazzo, Olatunde Aremu, Ashokan Arumugam, Alok Atreya, Avinash Aujayeb, Jose Luis Ayuso-Mateos, Luchuo Engelbert Bain, Maciej Banach, Till Winfried Bärnighausen, Francesco Barone-Adesi, Massimiliano Beghi, Derrick A. Bennett, Akshaya S. Bhagavathula, Félix Carvalho, Giulio Castelpietra, Ledda Caterina, Joht Singh Chandan, Rosa A. S. Couto, Natália Cruz-Martins, Giovanni Damiani, Anna Dastiridou, Andreas K. Demetriades, Diana Dias-da-Silva, Adeniyi Francis Fagbamigbe, Seyed-Mohammad Fereshtehnejad, Eduarda Fernandes, Pietro Ferrara, Florian Fischer, Urbano Fra.Paleo, Silvia Ghirini, James C. Glasbey, Ionela-Roxana Glavan, Nelson G. M. Gomes, Michal Grivna, Netanja I. Harlianto, Josep Maria Haro, M. Tasdik Hasan, Sorin Hostiuc, Ivo Iavicoli, Milena D. Ilic, Irena M. Ilic, Mihajlo Jakovljevic, Jost B. Jonas, Jacek Jerzy Jozwiak, Mikk Jürisson, Joonas H. Kauppila, Gbenga A. Kayode, Moien A. B. Khan, Adnan Kisa, Sezer Kisa, Ai Koyanagi, Manasi Kumar, Om P. Kurmi, Carlo La-Vecchia, Demetris Lamnisos, Savita Lasrado, Paolo Lauriola, Shai Linn, Joana A. Loureiro, Raimundas Lunevicius, Aurea Madureira-Carvalho, Enkeleint A. Mechili, Azeem Majeed, Ritesh G. Menezes, Alexios-Fotios A. Mentis, Atte Meretoja, Tomislav Mestrovic, Tomasz Miazgowski, Bartosz Miazgowski, Andreea Mirica, Mariam Molokhia, Shafiu Mohammed, Lorenzo Monasta, Francesk Mulita, Mukhammad David Naimzada, Ionut Negoi, Subas Neupane, Bogdan Oancea, Hans Orru, Adrian Otoiu, Nikita Otstavnov, Stanislav S. Otstavnov, Alicia Padron-Monedero, Songhomitra Panda-Jonas, Shahina Pardhan, Jay Patel, Paolo Pedersini, Marina Pinheiro, Ivo Rakovac, Chythra R. Rao, Salman Rawaf, David Laith Rawaf, Violet Rodrigues, Luca Ronfani, Dominic Sagoe, Francesco Sanmarchi, Milena M. Santric-Milicevic, Brijesh Sathian, Aziz Sheikh, Rahman Shiri, Siddharudha Shivalli, Inga Dora Sigfusdottir, Rannveig Sigurvinsdottir, Valentin Yurievich Skryabin, Anna Aleksandrovna Skryabina, Catalin-Gabriel Smarandache, Bogdan Socea, Raúl A. R. C. Sousa, Paschalis Steiropoulos, Rafael Tabarés-Seisdedos, Marcos Roberto Tovani-Palone, Fimka Tozija, Sarah Van de Velde, Tommi Juhani Vasankari, Massimiliano Veroux, Francesco S. Violante, Vasiliy Vlassov, Yanzhong Wang, Ali Yadollahpour, Sanni Yaya, Mikhail Sergeevich Zastrozhin, Anasthasia Zastrozhina, Suzanne Polinder, Marek Majdan

**Affiliations:** 1 Department of Public Health, Erasmus MC, University Medical Center, Rotterdam, The Netherlands; 2 Epidemiology Unit, Central Tuscany Local Health Authority, Florence, Italy; 3 Department of Non-Communicable Diseases and Injuries, Santé Publique France, Saint-Maurice, France; 4 Department of Public Health, Aarhus University, Aarhus, Denmark; 5 Laboratory of Social Medicine, Medical School, Democritus University of Thrace, Alexandroupolis, Greece; 6 School of Public Health and Community Medicine, Sahlgrenska Academy, University of Gothenburg, Gothenburg, Sweden; 7 Department of Epidemiology and Population Health, Faculty of Health Sciences, American University of Beirut, Beirut, Lebanon; 8 Department of Epidemiology and Health Monitoring, Robert Koch Institute, Berlin, Germany; 9 Institute of Hygiene, Health Information Center, Kaunas, Lithuania; 10 Department of Biostatistics, Near East University, Nicosia, Cyprus; 11 Department of Biostatistics and Health Informatics, Madda Walabu University, Bale Robe, Ethiopia; 12 Geriatrics and Long Term Care Department, Hamad Medical Corporation, Doha, Qatar; 13 Department of Experimental Medicine, University of Campania Luigi Vanvitelli, Naples, Italy; 14 Cardiology Department, Carol Davila University of Medicine and Pharmacy, Bucharest, Romania; 15 Department of Statistics and Econometrics, Bucharest University of Economic Studies, Bucharest, Romania; 16 Research Center On Public Health, University of Milan-Bicocca, Monza, Italy; 17 Department of Public Health, Birmingham City University, Birmingham, UK; 18 Department of Physiotherapy, College of Health Sciences, University of Sharjah, Sharjah, United Arab Emirates; 19 Department of Forensic Medicine, Lumbini Medical College, Palpa, Nepal; 20 Northumbria Healthcare NHS Foundation Trust, Cramlington, UK; 21 Hospital Universitario de La Princesa, Instituto de Investigación Sanitaria Princesa (IIS-Princesa), Madrid, Spain; 22 Carlos III Health Institute, Biomedical Research Networking Center for Mental Health Network (CiberSAM), Madrid, Spain; 23 Lincoln International Institute for Rural Health (LIIRH), University of Lincoln, Lincoln, UK; 24 Global South Health Services and Research, GSHS, Amsterdam, The Netherlands; 25 Department of Hypertension, Medical University of Lodz, Lodz, Poland; 26 Polish Mothers’ Memorial Hospital Research Institute, Lodz, Poland; 27 Heidelberg Institute of Global Health (HIGH), Heidelberg University, Heidelberg, Germany; 28 T.H. Chan School of Public Health, Harvard University, Boston, MA USA; 29 Department of Translational Medicine, University of Eastern Piedmont, Novara, Italy; 30 Department of Mental Health, AUSL Romagna, Cesena, Italy; 31 Clinical Trials Service Unit and Epidemiological Studies Unit, Nuffield Department of Population Health, University of Oxford, Oxford, UK; 32 Institute of Public Health, United Arab Emirates University, Al Ain, United Arab Emirates; 33 Department of Social and Clinical Pharmacy, Charles University, Hradec Kralova, Prague, Czech Republic; 34 Research Unit On Applied Molecular Biosciences (UCIBIO), University of Porto, Porto, Portugal; 35 Outpatient and Inpatient Care Service, Central Health Directorate, Friuli Venezia Giulia Region, Trieste, Italy; 36 Department of Clinical and Experimental Medicine, University of Catania, Catania, Italy; 37 Institute of Applied Health Research, University of Birmingham, Birmingham, UK; 38 Department of Chemical Sciences, University of Porto, Porto, Portugal; 39 Faculty of Medicine, University of Porto, Porto, Portugal; 40 Institute for Research & Innovation in Health (i3S), University of Porto, Porto, Portugal; 41 Institute of Research and Advanced, Training in Health Sciences and Technologies (INFACTS), Gandra, Portugal; 42 Clinical Dermatology, IRCCS Istituto Ortopedico Galeazzi, University of Milan, Milan, Italy; 43 Department of Dermatology, Case Western Reserve University, Cleveland, OH USA; 44 Department of Ophthalmology, University Hospital of Larissa, Larissa, Greece; 45 Edinburgh Spinal Surgery Outcome Studies Group, Department of Neurosurgery, Royal Infirmary of Edinburgh, Edinburgh, UK; 46 Laboratory of Toxicology, Faculty of Pharmacy, University of Porto, Porto, Portugal; 47 Department of Epidemiology and Medical Statistics, Faculty of Public Health, College of Medicine, University of Ibadan, Ibadan, Nigeria; 48 Division of Neurology, Department of Medicine, University of Ottawa, Ottawa, ON Canada; 49 Division of Clinical Geriatrics, Department of Neurobiology, Karolinska Institute, Stockholm, Sweden; 50 Associated Laboratory for Green Chemistry (LAQV), University of Porto, Porto, Portugal; 51 Institute of Public Health, Charité-Universitätsmedizin Berlin, Berlin, Germany; 52 Research Institute for Sustainable Land Development (Interra), University of Extremadura, Caceres, Spain; 53 National Center On Addictions and Doping, Istituto Superiore Di Sanità, Rome, Italy; 54 NIHR Global Health Research Unit On Global Surgery, University of Birmingham, Birmingham, UK; 55 Department of Chemistry, University of Porto, Porto, Portugal; 56 Department of Orthopedics, University Medical Center Utrecht, Utrecht, Netherlands; 57 Biomedical Research Networking Center for Mental Health Network (CiberSAM), Madrid, Spain; 58 Research and Development Unit, San Juan de Dios Sanitary Park, Sant Boi de Llobregat, Spain; 59 International Centre for Diarrhoeal Disease Research, Dhaka, Bangladesh; 60 Department of Primary Care and Mental Health, University of Liverpool, Liverpool, UK; 61 Department of Legal Medicine and Bioethics, Carol Davila University of Medicine and Pharmacy, Bucharest, Romania; 62 Clinical Legal Medicine Department, National Institute of Legal Medicine Mina Minovici, Bucharest, Romania; 63 Department of Public Health, University of Naples Federico II, Naples, Italy; 64 Department of Epidemiology, University of Kragujevac, Kragujevac, Serbia; 65 Faculty of Medicine, University of Belgrade, Belgrade, Serbia; 66 N. A. Semashko Department of Public Health and Healthcare, I. M. Sechenov First Moscow State Medical University, Moscow, Russia; 67 Department of Global Health, Economics and Policy, University of Kragujevac, Kragujevac, Serbia; 68 Department of Ophthalmology, Heidelberg University, Mannheim, Germany; 69 Beijing Institute of Ophthalmology, Beijing Tongren Hospital, Beijing, China; 70 Department of Family Medicine and Public Health, University of Opole, Opole, Poland; 71 Institute of Family Medicine and Public Health, University of Tartu, Tartu, Estonia; 72 Department of Molecular Medicine and Surgery, Karolinska Institute, Stockholm, Sweden; 73 Surgery Research Unit, University of Oulu, Oulu, Finland; 74 International Research Center of Excellence, Institute of Human Virology Nigeria, Abuja, Nigeria; 75 Julius Centre for Health Sciences and Primary Care, Utrecht University, Utrecht, The Netherlands; 76 Department of Family Medicine, United Arab Emirates University, Al-Ain, United Arab Emirates; 77 Primary Care Department, NHS North West London, London, England; 78 School of Health Sciences, Kristiania University College, Oslo, Norway; 79 Department of Global Community Health and Behavioral Sciences, Tulane University, New Orleans, LA USA; 80 Department of Nursing and Health Promotion, Oslo Metropolitan University, Oslo, Norway; 81 Biomedical Research Networking Center for Mental Health Network (CiberSAM), San Juan de Dios Sanitary Park, Sant Boi de Llobregat, Spain; 82 Catalan Institution for Research and Advanced Studies (ICREA), Barcelona, Spain; 83 Department of Psychiatry, University of Nairobi, Nairobi, Kenya; 84 Division of Psychology and Language Sciences, University College London, London, UK; 85 Division of Respirology, Department of Medicine, McMaster University, Hamilton, Canada; 86 Faculty of Health and Life Sciences, Coventry University, Coventry, UK; 87 Department of Clinical Sciences and Community Health, University of Milan, Milan, Italy; 88 Department of Health Sciences, School of Sciences, European University Cyprus, Nicosia, Cyprus; 89 Department of Otorhinolaryngology, Father Muller Medical College, Mangalore, India; 90 Institute of Clinical Physiology, National Research Council, Pisa, Italy; 91 School of Public Health, University of Haifa, Haifa, Israel; 92 Laboratory for Process Engineering, Environment, Biotechnology and Energy (LEPABE), University of Porto, Porto, Portugal; 93 Department of General Surgery, School of Medicine, Liverpool University Hospitals NHS Foundation Trust, University of Liverpool, Liverpool, UK; 94 Laboratório de Farmacognosia, Departamento de Química, Faculdade de Farmácia, Universidade Do Porto, Porto, Portugal; 95 Clinic of Social and Family Medicine, School of Medicine, University of Crete, Crete, Greece; 96 Department of Healthcare, Faculty of Public Health, University of Vlora, Vlora, Albania; 97 Department of Primary Care and Public Health, Imperial College London, London, UK; 98 Forensic Medicine Division, Imam Abdulrahman Bin Faisal University, Dammam, Saudi Arabia; 99 Public Health Laboratories, Hellenic Pasteur Institute, Athens, Greece; 100 Department of Neurology, University Hospital of Larissa, University of Thessaly, Larissa, Greece; 101 School of Health Sciences, University of Melbourne, Melbourne, VIC Australia; 102 Neurology Unit, Helsinki University Hospital, Helsinki, Finland; 103 Clinical Microbiology and Parasitology Unit, Dr Zora Profozic Polyclinic, Zagreb, Croatia; 104 University Centre Varazdin, University North, Varazdin, Croatia; 105 Department of Propedeutics of Internal Diseases & Arterial Hypertension, Pomeranian Medical University, Szczecin, Poland; 106 Center for Innovation in Medical Education, Pomeranian Medical University, Szczecin, Poland; 107 Faculty of Life Sciences and Medicine, King’s College London, London, UK; 108 Health Systems and Policy Research Unit, Ahmadu Bello University, Zaria, Nigeria; 109 Clinical Epidemiology and Public Health Research Unit, Burlo Garofolo Institute for Maternal and Child Health, Trieste, Italy; 110 Department of General Surgery, University General Hospital of Patras, Patras, Greece; 111 Laboratory of Public Health Indicators Analysis and Health Digitalization, Moscow Institute of Physics and Technology, Dolgoprudny, Russia; 112 Experimental Surgery and Oncology Laboratory, Kursk State Medical University, Kursk, Russia; 113 Department of General Surgery, Carol Davila University of Medicine and Pharmacy, Bucharest, Romania; 114 Department of General Surgery, Emergency Hospital of Bucharest, Bucharest, Romania; 115 Faculty of Social Sciences, Unit of Health Sciences, Tampere University, Tampere, Finland; 116 Gerontology Research Center, Tampere University, Tampere, Finland; 117 Administrative and Economic Sciences Department, University of Bucharest, Bucharest, Romania; 118 Department of Public Health and Clinical Medicine, Sustainable Health, Umea University, Umea, Sweden; 119 Department of Project Management, National Research University Higher School of Economics, Moscow, Russia; 120 National School of Public Health, Institute of Health Carlos III, Madrid, Spain; 121 Vision and Eye Research Institute, Anglia Ruskin University, Cambridge, UK; 122 Faculty of Medicine and Health, University of Leeds, Leeds, UK; 123 IRCCS Fondazione Don Carlo Gnocchi, Milan, Italy; 124 World Health Organization (WHO) European Office for the Prevention and Control of Noncommunicable Diseases, Division of Country Health Programmes, WHO Regional Office for Europe, Moscow, Russian Federation; 125 Department of Community Medicine, Kasturba Medical College, Manipal Academy of Higher Education, Manipal, Karnataka India; 126 Academic Public Health Department, Public Health England, London, UK; 127 World Health Organization (WHO) Collaborating Centre for Public Health Education and Training, Imperial College London, London, UK; 128 University College London Hospitals, London, UK; 129 Community Nursing Unit, Ireland Hospital, Abbeyleix, Ireland; 130 Department of Psychosocial Science, University of Bergen, Bergen, Norway; 131 Department of Biomedical and Neuromotor Sciences, Alma Mater Studiorum, University of Bologna, Bologna, Italy; 132 School of Public Health and Health Management, University of Belgrade, Belgrade, Serbia; 133 Faculty of Health & Social Sciences, Bournemouth University, Bournemouth, UK; 134 Centre for Medical Informatics, University of Edinburgh, Edinburgh, UK; 135 Division of General Internal Medicine, Harvard University, Boston, MA USA; 136 Finnish Institute of Occupational Health, Helsinki, Finland; 137 Department of Medical Statistics, London School of Hygiene & Tropical Medicine, London, UK; 138 Department of Psychology, Reykjavik University, Reykjavik, Iceland; 139 Icelandic Centre for Social Research and Analysis (ICSRA), Reykjavik, Iceland; 140 Department of Health and Behavior Studies, Teachers College, Columbia University, New York, NY USA; 141 Department No.16, Moscow Research and Practical Centre On Addictions, Moscow, Russia; 142 Therapeutic Department, Balashiha Central Hospital, Balashikha, Russia; 143 Professional Association of Licensed Optometry Professionals, Linda-a-Velha, Portugal; 144 Department of Respiratory Medicine, Medical School, Democritus University of Thrace, University General Hospital Dragana, Alexandroupolis, Greece; 145 Department of Medicine, University of Valencia, Valencia, Spain; 146 Department of Pathology and Legal Medicine, University of São Paulo, Ribeirão Preto, Brazil; 147 Institute of Public Health of Republic of North Macedonia, Saints Cyril and Methodius University of Skopje, Skopje, North Macedonia; 148 Centre for Population, Family and Health, Department of Sociology, University of Antwerp, Antwerp, Belgium; 149 UKK Institute, Tampere, Finland; 150 Department of Medical, Surgical Sciences and Advanced Technologies, University of Catania, Catania, Italy; 151 Department of Medical and Surgical Sciences, University of Bologna, Bologna, Italy; 152 Occupational Health Unit, Sant’Orsola Malpighi Hospital, Bologna, Italy; 153 Department of Health Care Administration and Economics, National Research University Higher School of Economics, Moscow, Russia; 154 School of Population Health and Environmental Sciences, King’s College London, London, UK; 155 Psychology Department, University of Sheffield, Sheffield, UK; 156 School of International Development and Global Studies, University of Ottawa, Ottawa, ON Canada; 157 The George Institute for Global Health, University of Oxford, Oxford, UK; 158 Laboratory of Genetics and Genomics, Moscow Research and Practical Centre On Addictions, Moscow, Russia; 159 Addictology Department, Russian Medical Academy of Continuous Professional Education, Moscow, Russia; 160 Pediatrics Department, Russian Medical Academy of Continuous Professional Education, Moscow, Russia; 161 Department of Public Health, Institute for Global Health and Epidemiology, Faculty of Health Sciences and Social Work, Trnava University, Trnava, Slovakia

**Keywords:** Burden of disease, Injuries, Disability adjusted life years, Mortality, Europe

## Abstract

**Background:**

Injury remains a major concern to public health in the European region. Previous iterations of the Global Burden of Disease (GBD) study showed wide variation in injury death and disability adjusted life year (DALY) rates across Europe, indicating injury inequality gaps between sub-regions and countries. The objectives of this study were to: 1) compare GBD 2019 estimates on injury mortality and DALYs across European sub-regions and countries by cause-of-injury category and sex; 2) examine changes in injury DALY rates over a 20 year-period by cause-of-injury category, sub-region and country; and 3) assess inequalities in injury mortality and DALY rates across the countries.

**Methods:**

We performed a secondary database descriptive study using the GBD 2019 results on injuries in 44 European countries from 2000 to 2019. Inequality in DALY rates between these countries was assessed by calculating the DALY rate ratio between the highest-ranking country and lowest-ranking country in each year.

**Results:**

In 2019, in Eastern Europe 80 [95% uncertainty interval (UI): 71 to 89] people per 100,000 died from injuries; twice as high compared to Central Europe (38 injury deaths per 100,000; 95% UI 34 to 42) and three times as high compared to Western Europe (27 injury deaths per 100,000; 95%UI 25 to 28). The injury DALY rates showed less pronounced differences between Eastern (5129 DALYs per 100,000; 95% UI: 4547 to 5864), Central (2940 DALYs per 100,000; 95% UI: 2452 to 3546) and Western Europe (1782 DALYs per 100,000; 95% UI: 1523 to 2115). Injury DALY rate was lowest in Italy (1489 DALYs per 100,000) and highest in Ukraine (5553 DALYs per 100,000). The difference in injury DALY rates by country was larger for males compared to females. The DALY rate ratio was highest in 2005, with DALY rate in the lowest-ranking country (Russian Federation) 6.0 times higher compared to the highest-ranking country (Malta). After 2005, the DALY rate ratio between the lowest- and the highest-ranking country gradually decreased to 3.7 in 2019.

**Conclusions:**

Injury mortality and DALY rates were highest in Eastern Europe and lowest in Western Europe, although differences in injury DALY rates declined rapidly, particularly in the past decade. The injury DALY rate ratio of highest- and lowest-ranking country declined from 2005 onwards, indicating declining inequalities in injuries between European countries.

## Background

An injury is defined as any intentional or unintentional bodily harm that results from tissue damage due to acute exposure to energy (mechanical, thermal, electrical, chemical, radiation), or cellular death, or loss of homeostasis [1]. Injuries are recognized as a major concern in public health worldwide. Results of the Global Burden of Disease (GBD) study showed that globally in 2019, 8% of all deaths were due to injury [2]. In the European region, the share of injury deaths was 5% [3]; however, major differences across European countries are observed, ranging from a low of 3% in Bulgaria to a high of 8% in Russia.

Apart from a major cause of death, injury is also often cited as an important cause of disability. Cohort studies among trauma patients showed that the majority of trauma patients had lower health-related quality of life scores one year after sustaining the injury, compared to their pre-injury health status or the general population [4, 5]. Only a share of patients with long-term consequences of injury will recover, whereas most will experience permanent disabilities [6–8]. These findings highlight the importance of including both fatal and non-fatal consequences of injury, when describing the population health impact of injury.

A widely used population health metric that incorporates the years of life lost due to premature mortality (YLL) and years lived with disability (YLD) is the disability adjusted life year (DALY) [9]. This composite measure allows comparison of the population health impact of diseases and injuries with varying incidence and case fatality rates. By calculating age-standardized DALY rates, the DALYs are adjusted for differences in age structure and size of the populations. Hence, population health impact of different causes of disease and injury can be compared across countries and over time.

Comparisons of the population health impact of different causes of injury are crucial for the identification of major causes of injury and injury DALY trends over time, which may serve as input for priority-setting with regards to national injury prevention measures and their effects and health service planning [10]. Moreover, comparison of injury DALY rates may help to identify the existence of health inequality gaps between countries. Health inequality gaps are unfair differences in health status between sub-groups of a population that are avoidable [11]. Injuries are highly preventable [12], but prevention of injury requires material, economic or social means to protect oneself or others in the community. However, injuries are not equally distributed within societies and subsequently may result in health inequalities that can be measured by differences in injury incidence and mortality rates across populations [13, 14]. A recently published systematic review on inequalities in injuries in the European region identified two cross-country studies that investigated inequalities over time [14]. Both studies were limited to children aged 1 to 14 years and used mortality rate ratios to investigate inequalities in injuries, instead of an integrative measure that includes both fatal and non-fatal outcomes, such as the DALY [15, 16]. Insight into health inequalities in injuries across countries and within populations, using the DALY metric is currently lacking in Europe.

Therefore, the objectives of this study were to: 1) compare the GBD 2019 estimates on injury mortality and DALYs across 44 countries of the GBD European region (i.e., Central, Eastern, and Western Europe) by cause-of-injury category and sex; 2) examine changes in injury DALY over a 20 year-period by cause-of-injury category, sub-region and country; and 3) assess inequalities in injury mortality and DALY rates across Central, Eastern, and Western European countries.

## Methods

We analyzed levels and trends of incidence, mortality, and DALY and its components: YLL and YLD of injury in the European region of the GBD 2019 study [2]. The DALY is calculated by adding YLLs and YLDs. YLLs are calculated by multiplying deaths by the remaining life expectancy at the age of death. YLDs are calculated by multiplying the number of cases with a certain health outcome with the disability weight assigned to this health outcome. One DALY is equivalent to one healthy life year lost from mortality and disability.

The GBD 2019 study provided global and regional estimates for 286 causes of death, 369 diseases and injuries, for 23 age groups, male and female sex, and for 204 countries and territories from 1990 to 2019 [2]. Detailed descriptions of the methodology and approach of the GBD study and supplemental information on methods that were used to calculate incidence, mortality, YLL, YLD and DALY estimates have been published elsewhere [1, 2]. For the present study, we used the GBD 2019 interactive data visualization tool ‘GBD Compare’ to retrieve the estimates for injury incidence, mortality, YLLs, YLDs, and DALYs (GBD 2019 Results. Seattle, United States: Institute for Health Metrics and Evaluation (IHME), 2019; http://vizhub.healthdata.org/gbd-compare/). In our study, we used estimates for each year in the period between 2000 and 2019. We compared incidence, mortality, YLL, YLD, and DALY by sex, country, and over time.

### Cause-of-injury categories

Injury incidence and mortality data, coded according to the International Classification of Diseases, Ninth Revision (ICD-9) and the International Statistical Classification of Diseases and Related Health Problems, 10th Revision (ICD-10), were categorized into mutually exclusive and collectively exhaustive GBD cause-of-injury categories [1]. The cause-of-injury categories covered by the GBD were arranged in standard hierarchical categories of four levels. Level 1 causes consist of the category “Injuries” (Group III). This level can be broken down into three Level 2 cause-of-injury classifications, namely “Unintentional injury”, “Transport injury” and “Self-harm and interpersonal violence”. These level 2 causes can be further broken down into seventeen Level 3 and twenty-four Level 4 cause-of-injury categories. The Level 4 cause-of-injury categories convey the most detail about the causes of injury. For example, the Level 2 cause-of-injury category “Self-harm and interpersonal violence” is subdivided into Level 3 cause-of-injury categories “Self-harm” and “Interpersonal violence”. The Level 3 cause-of injury-category “Interpersonal violence” can be broken down into four Level 4 categories “Psychical violence by firearm”, “Psychical violence by sharp object”, “Psychical violence by other means” and “Sexual violence”. The case definitions and ICD-codes of each of the cause-of-injury categories used in the GBD 2019 study can be found elsewhere [1, 2]. For the present analysis, we report the Level 3 cause-of-injury categories. Injury incidence was restricted to cases warranting some form of healthcare, including General Practitioner and Emergency Department visits, in a healthcare system, where patients have full, unrestricted access to healthcare.

### Selection of countries

In GBD 2019, Europe is divided into three regions: the Central European region (13 countries), the Eastern European region (7 countries) and the Western European region (24 countries). Thirteen countries were included in the Central European region of the GBD: Albania, Bosnia and Herzegovina, Bulgaria, Croatia, Czechia, Hungary, North Macedonia, Montenegro, Poland, Romania, Serbia, Slovakia and Slovenia. Seven countries were included in the Eastern European region of the GBD: Belarus, Estonia, Latvia, Lithuania, Republic of Moldova, Russian Federation and Ukraine. Twenty-four countries were included in the Western European region of the GBD: Andorra, Austria, Belgium, Cyprus, Denmark, Finland, France, Germany, Greece, Iceland, Ireland, Israel, Italy, Luxembourg, Malta, Monaco, Netherlands, Norway, Portugal, San Marino, Spain, Sweden, Switzerland and United Kingdom.

### Percent change

The percent change over the 2000–2019 period is calculated by subtracting the DALY estimates for the year 2000 from the DALY estimate for the year 2019 and dividing it by the DALY estimate of the reference starting-point (i.e., the year 2000). A positive change indicates an increase of the burden resulting from that specific cause-of-injury during the 20-year study period, whereas a negative change indicates a decrease.

### Assessment of inequality in mortality and DALY rates

Inequality in mortality rate between these 44 countries was calculated using the ratio of mortality rate for the highest-ranking country according to injury mortality rates to lowest-ranking country in each year. Inequality in DALY rate between countries was calculated using the ratio of DALY rate for the highest-ranking country according to injury DALY rates to lowest-ranking country in each year.

### Uncertainty

The GBD estimates have varying degrees of uncertainty in the input data, the data adjustments, and the statistical models used to estimate values for all geographical locations over time [1, 2]. Standard GBD methodology is that for each outcome variable (incidence, mortality, YLL, YLD, and DALY), uncertainty from each source is propagated at the level of 1000 draws; that is, all estimates were calculated 1000 times, each time drawing from the posterior distributions. In the Results section, we present the median value of the 1000 draws of the sampled incidence, mortality, YLL, YLD, and DALY values. We also present the 95% uncertainty interval (UI), which corresponds to the 2.5th and 97.5th percentiles of the corresponding distribution.

## Results

### Age-standardized incidence rates of injuries by European sub-region, 2019

Table 1 shows the incidence and mortality rates by all causes of injury and by European sub-region. The age-standardized incidence rates per 100,000 varied between Central, Eastern, and Western Europe. In 2019 in Central Europe, we observed 22,527 (95% UI: 20,338 to 24,899) new cases per 100,000, while incidence rates of all causes of injury in Eastern and Western Europe were 18,983 (95% UI: 17,295 to 20,784) and 12,313 (95%UI 11,049 to 13,739) per 100,000, respectively. Between 2000 and 2019, the change in incidence rates for all injuries has been decreased only by -3.3% (Central Europe) and -3.5% (Western Europe), and by -18.9% in Eastern Europe. Over the same period, falls and exposure to mechanical forces tend to be the highest incident causes of injury across all the European regions.

**Table 1:** Incidence and mortality rates by cause of injury (Level 3) and by European sub-region with 95% uncertainty interval, 2019

Cause of injury	Mortality rate (per 100,000)			Incidence rate (per 100,000)		
	Central Europe	Eastern Europe	Western Europe	Central Europe	Eastern Europe	Western Europe
All causes of injury	37.9 (33.5 – 42.4)	80.1 (71.4 – 89.2)	26.7 (25.0 – 28.0)	22,527.5 (20,338.1 – 24,899.4)	18,983.2 (17,294.7 – 20,783.7)	12,313 (11,049.4 – 13,738.9)
Road injuries	7.9 (7.0 – 9.0)	13.3 (11.9 – 15)	4.9 (4.7 – 5.1)	1901 (1632 – 2194)	2600 (2123 – 3143)	522 (447 – 612)
Other transport injuries	1.1 (1.0 – 1.2)	1.4 (1.2 – 1.6)	0.5 (0.5 – 0.6)	52.2 (40.1 – 67.0)	53.4 (40.9 – 69.4)	35.7 (27.7 – 46.5)
Falls	8.0 (7.0 – 9.0)	6.40 (5.8 – 7.1)	7.4 (6.5 – 7.9)	6674.5 (5642.8 – 7824.9)	6026.2 (5047.2 – 7185.2)	5841.7 (4886.3 – 6998.5)
Drowning	1.7 (1.5 – 2.0)	5.1 (4.6 – 5.7)	0.65 (0.6 – 0.7)	12.5 (10.5 – 15.1)	15.8 (13.1 – 19.0)	5.5 (4.5 – 6.6)
Fire, heat, and hot substances	0.9 (0.8 – 1.0)	3.5 (3.1 – 3.9)	0.4 (0.4 – 0.5)	302.0 (227.6 – 375.7)	258.0 (195.0 – 324.3)	164.9 (122.2 – 208.1)
Poisonings	0.5 (0.5 – 0.5)	3.08 (2.7 – 3.4)	0.15 (0.14 – 0.15)	151.5 (110.2 – 203.5)	128.5 (95.1 – 170.3)	73.8 (54.5 – 96.9)
Exposure to mechanical forces	0.8 (0.7 – 0.9)	1.7 (1.5 – 1.9)	0.4 (0.4 – 0.4)	8863.7 (6999.5 – 10,903.4)	5198.7 (4121.1 – 6310.7)	2841.4 (2155.9 – 3547.8)
Adverse effects of medical treatment	0.7 (0.5 – 0.8)	0.7 (0.5 – 0.9)	1.0 (0.9 – 1.1)	333.9 (271.1 – 403.2)	241.5 (195.2 – 296.4)	205.8 (169.1 – 251.6)
Animal contact	0.09 (0.08 – 0.1)	0.14 (0.12 – 0.16)	0.04 (0.04 – 0.04)	916.0 (695.6 – 1250.8)	716.4 (543.5 – 979.6)	275.8 (207.7 – 381.0)
Foreign body	1.5 (1.3 – 1.7)	3.5 (3.1 – 3.9)	1.3 (1.2 – 1.4)	924.6 (758.8 – 1151.5)	1042.1 (846.1 – 1310.5)	674.8 (555.7 – 832.4)
Other unintentional injuries	0.7 (0.6 – 0.8)	1.8 (1.6 – 2.0)	0.2 (0.2 – 0.2)	1741.3 (1362.0 – 2166.2)	1481.4 (1170.8 – 1829.2)	1245.7 (957.5 – 1573.1)
Self-harm	11.5 (10.0 – 13.2)	23.05 (20.2 – 26.9)	8.5 (8.1 – 8.9)	80.8 (71.9 – 90.9)	161.1 (133.9 – 196.1)	67.6 (61.2 – 75.5)
Interpersonal violence	1.5 (1.3 – 1.6)	11. 7 (10.4 – 13.2)	0.75 (0.7 – 0.8)	531.8 (408.0 – 661.4)	757.8 (588.6 – 939.2)	294.4 (220.2 – 371.7)
Exposure to forces of nature	0.04 (0.04 – 0.05)	0.00 (0.00 – 0.00)	0.00 (0.00 – 0.00)	0.00 (0.00 – 0.00)	0.00 (0.00 – 0.00)	0.1 (0.1 – 0.2)
Environmental heat and cold exposure	0.8 (0.7 – 0.9)	4.5 (4.0 – 5.1)	0.36 (0.34 – 0.39)	41.3 (32.7 – 53.1)	280.7 (221.8 – 356.7)	62.9 (44.7 – 88.5)
Conflict and terrorism	0.00 (0.00 – 0.00)	0.12 (0.11 – 0.13)	0.00 (0.00 – 0.00)	0.00 (0.00 – 0.00)	19.4 (15.7 – 23.3)	0.4 (0.3 – 0.5)
Police conflict and executions	0.01 (0.01 – 0.01)	0.07 (0.06 – 0.08)	0.01 (0.01 – 0.01)	0.00 (0.00 – 0.00)	1. 9 (2.4 – 1.4)	0.00 (0.00 – 0.00)

### Age-standardized injury mortality rates by European sub-region, 2019

In 2019, in all European countries taken together, the incidence of all-cause injury was 109.7 million and 458,669 people died from injuries. The injury mortality rate per 100,000 individuals varied between European sub-regions. In Eastern Europe, 80 (95% UI: 71.4 to 89.2) individuals per 100,000 died from injuries; twice as high compared to Central Europe (injury deaths 37.8 per 100,000; 95% UI: 33.5 to 42.3) and almost three times as high compared to Western Europe (26.7 injury deaths per 100,000; 95% UI: 25.2 to 27.6). In Eastern Europe self-harm, road injuries and interpersonal violence contributed the most to the injury mortality rate (see Table 1). In Central and Western Europe, the causes of injury that contributed the most to the injury mortality rate were self-harm, road injuries, and falls. The highest variation in mortality rates by cause-of-injury death between European sub-regions was observed for poisonings (21 times higher in Eastern Europe compared to Western Europe), interpersonal violence (16 times higher in Eastern Europe compared to Western Europe) and environmental cold and heat exposure (13 times higher in Eastern Europe compared to Western Europe).

### Age-standardized injury DALY rates by European sub-region, 2019

Table 2 shows the DALY rates per 100,000 by cause-of-injury category and by European sub-region. The injury DALY rate per 100,000 was highest in the Eastern European region (5129 DALYs per 100,000; 95% UI: 4547 to 5864), followed by the Central European region (2940 DALYs per 100,000; 95% UI: 2452 to 3546) and the Western European region (1782 DALYs per 100,000; 95% UI: 1523 to 2115). In Eastern Europe, self-harm (1117 DALYs per 100,000; 95% UI: 980.5 to 1299) and road injuries (1061 DALYs per 100,000; 95% UI: 928 to 1226) contributed most to the injury DALY rate. In Central Europe, falls (706 DALYs per 100,000; 95% UI: 543 to 931) and road injuries (648 DALYs per 100,000; 95% UI: 551 to 754) contributed the most to the injury DALY rate, whereas in Western Europe the major contributors to injury DALY rates were falls (580 DALYs per 100,00; 95% UI: 440 to 768) and self-harm (372 DALYs per 100,000; 95% UI: 360 to 391).

**Table 2:** DALY rates and per cent change in DALYs 2000–2019 by cause of injury (Level 3) and by European sub-region with 95% uncertainty interval, 2019

Cause of injury	DALY rate (per 100,000)			Per cent of change (%)^a^ (2000–2019)		
	Central Europe	Eastern Europe	Western Europe	Central Europe	Eastern Europe	Western Europe
All causes of injury	2940.1 (2452.3 – 3546.2)	5129.2 (4547.3 – 5864)	1781. 9 (1523.1 – 2115.5)	-28.8	-44.6	-27.0
Road injuries	648.2 (551.5 – 754.0)	1061.3 (928.4 – 1226.4)	314.6 (291.2 – 341.2)	-36.6	-35.0	-55.6
Other transport injuries	60.9 (54.1 – 68.6)	78.6 (68.4 – 93.1)	33.4 (30.8 – 36.4)	-32.3	-8.6	-23.9
Falls	706.3 (542.8 – 931.2)	712.9 (566.8 – 924.1)	580.5 (440.4 – 768.2)	-9.6	-29.1	0.5
Drowning	88.7 (78.7 – 99.9)	273.8 (247.2 – 300.9)	32.1 (30.4 – 33.8)	-51.6	-61.5	-40.3
Fire, heat, and hot substances	86.7 (65.1 – 118.9)	188.9 (164.9 – 220.2)	45.0 (32.4 – 62.3)	-30.0	-55.3	-26.5
Poisonings	43.6 (35.0 – 53.2)	149.9 (133.8 – 165.9)	16.3 (12.7 – 20.6)	-49.0	-56.7	-24.9
Exposure to mechanical forces	357.4 (247.4 – 513.8)	265.5 (201.4 – 355.8)	122.6 (85.6 – 174.7)	-9.5	-32.3	-16.0
Adverse effects of medical treatment	23.1 (18.1 – 26.7)	27.6 (21.5 – 31.4)	25.5 (22.5 – 27.7)	-13.1	-12.4	-10.8
Animal contact	14.8 (10.8 – 20.0)	14.9 (11.9 – 18.9)	4.5 (3.5 – 6.0)	-19.5	-26.9	-16.2
Foreign body	89.5 (77.7 – 101.4)	189.5 (169.4 – 209.4)	52.6 (46.9 – 59.4)	-33.8	-39.2	-22.3
Other unintentional injuries	148.2 (106.0 – 205.5)	184.5 (149.0 – 235.4)	89.0 (60.2 – 130.0)	-37.5	-38.5	-18.4
Self-harm	508.3 (444.0 – 578.1)	1117.3 (980.5 – 1298.8)	372.2 (359.8 – 390.7)	-28.4	-40.3	-24.9
Interpersonal violence	117.5 (103.0 – 134.1)	633.5 (562.0 – 712.8)	72.2 (64.1 – 81.9)	-46.2	-55.0	-28.4
Exposure to forces of nature	2.6 (2.3 – 2.8)	0.3 (0.2 – 0.4)	0.4 (0.3 – 0.5)	39.4	-89.5	-83.6
Environmental heat and cold exposure	31.2 (27.3 – 35.4)	209.0 (185.8 – 234.2)	18.1 (15.7 – 20.9)	-35.5	-59.8	45.9
Conflict and terrorism	12.7 (8.3 – 20.3)	17.9 (13.8 – 24.9)	2.3 (1.5 – 3.7)	-76.0	-90.3	-53.7
Police conflict and executions	0.7 (0.6 – 0.8)	3.7 (3.2 – 4.2)	0.7 (0.6 – 0.7)	-14.1	-20.5	0.1

^a^The percent of change is the percentage change in DALY rate in the period from 2000 to 2019. A positive percentage of change indicates an increase; a negative percentage of change indicates a decrease

Highest variation in injury DALY rates between the European sub-regions was observed for environmental heat and cold exposure (12 times higher in Eastern Europe compared to Western Europe) and interpersonal violence, poisoning and drowning (all 9 times higher in Eastern Europe compared to Western Europe).

### Age standardized injury DALY rates by country, 2019

**Fig. 1:**
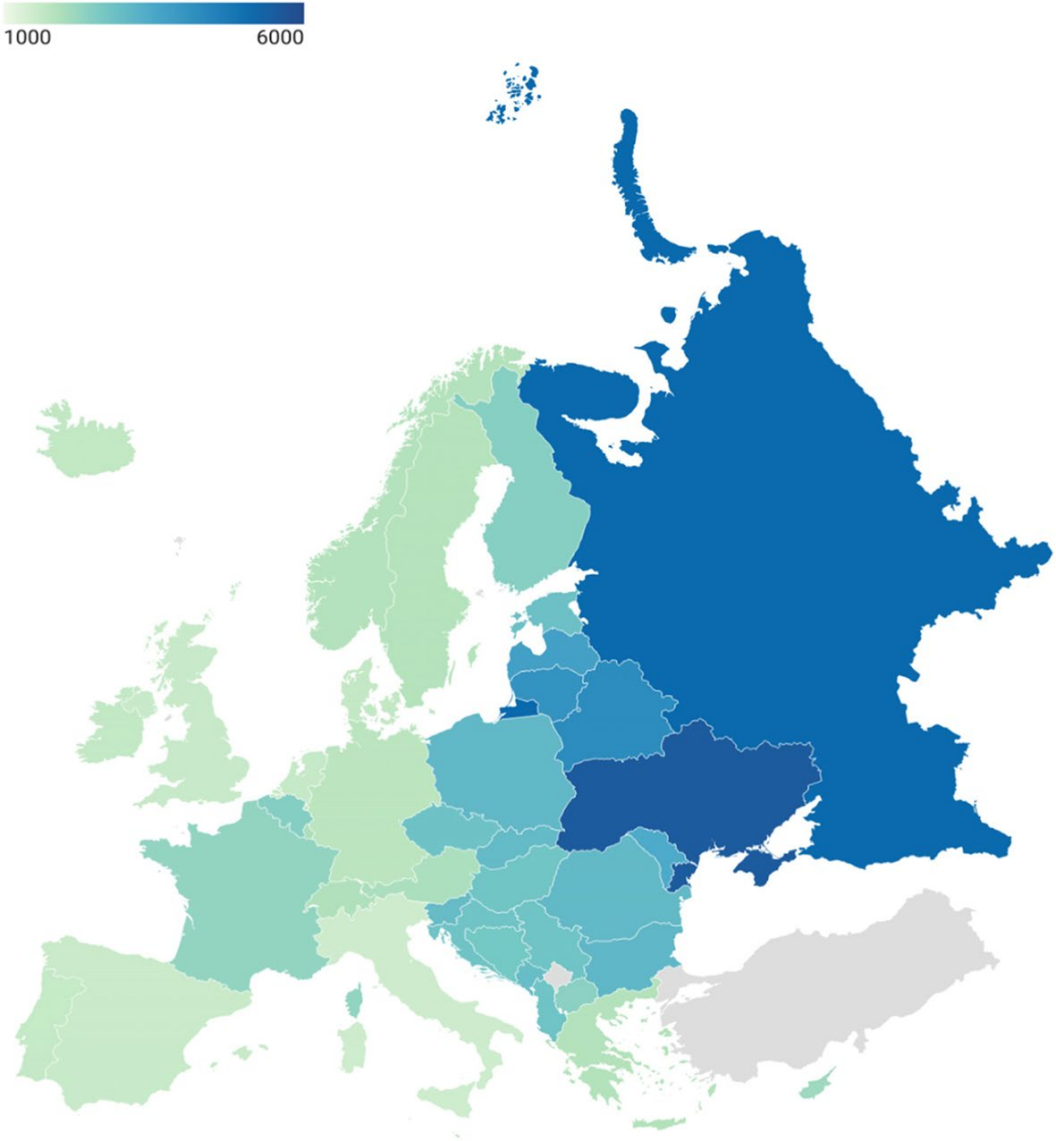
Map figure with age-standardised DALY rate of injury per 100,000 per country, 2019. *Countries in grey indicate that they do not belong to the GBD European sub-regions

Figure 1 shows the age-standardized DALY rate of injury per 100,000 per country. Injury DALY rates were lowest in Italy (1489 DALYs per 100,000; 95% UI: 1272 to 1764), Spain (1568 DALYs per 100,000; 95% UI: 1323 to 1887) and United Kingdom (1575 per 100,000; 95% UI: 1333 to 1898) and highest in Belarus (4264 DALYs per 100,000; 95% UI: 3489 to 5231), Russian Federation (5163 DALYs per 100,000; 95% UI: 4507 to 5954) and Ukraine (5553 DALYs per 100,000; 95% UI: 4784 to 6401).

**Fig. 2:**
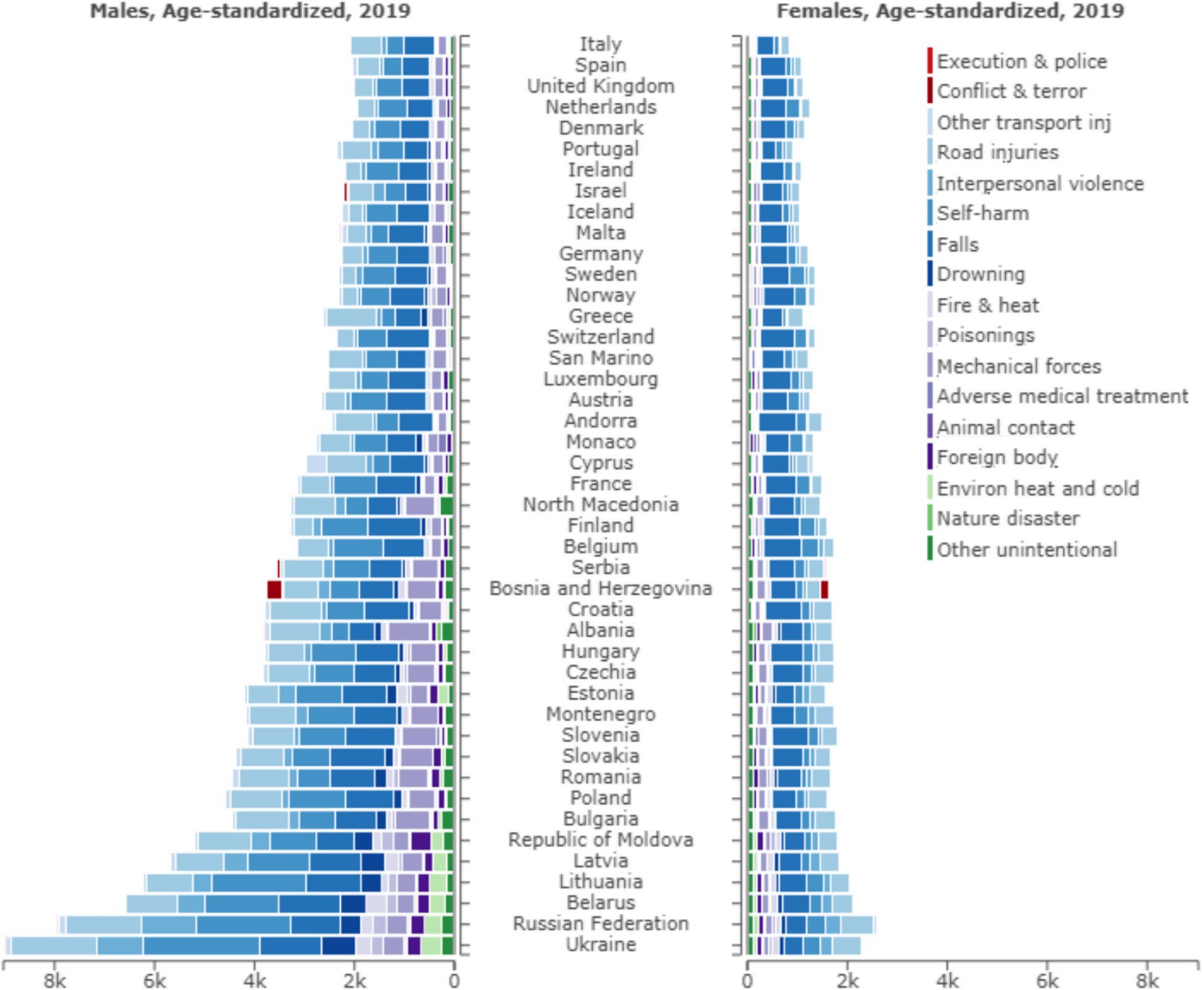
Pyramid figure with DALY rate by sex, country and cause of injury (Level 3), 2019

Figure 2 shows the DALY rates per 100,000 by cause-of-injury category, by sex, and by country for 2019. Across all the European region countries, injury rates were higher in males than females. For males, DALY rates per 100,000 varied from a high of 9024 (95% UI: 7680 to 10,582) in Ukraine to a low of 1952 (95% UI: 1689 to 2290) in the Netherlands, whereas in females DALY rates varied from a high of 2587 (95% UI: 2173 to 3097) in the Russian Federation to a low of 866 (95% UI: 713 to 1054) in Italy. In females, the DALY rates are driven by falls, with highest falls DALY rates in Belgium (751 DALYs per 100,000; 95% UI: 558 to 998), Finland (747 DALYs per 100,000; 95% UI: 542 to 1008), and Slovenia (731 DALYs per 100,000; 95% UI: 538 to 978). However, in Ukraine and the Russian Federation, highest DALY rates in females were observed for road injury rather than falls. In males, falls, self-harm and road injuries were the most prominent causes of injury in the countries with lowest injury DALY rates. The DALY rate due to exposure to mechanical forces were far higher in Romania, Slovakia, Bulgaria, and Albania compared to other European countries. Moreover, in countries with the highest injury DALY rates in males (Republic of Moldova, Latvia, Lithuania, Belarus, the Russian Federation and Ukraine) the DALY rates due to self-harm stand out.

### Changes in DALY rates, 2000 – 2019

**Fig. 3:**
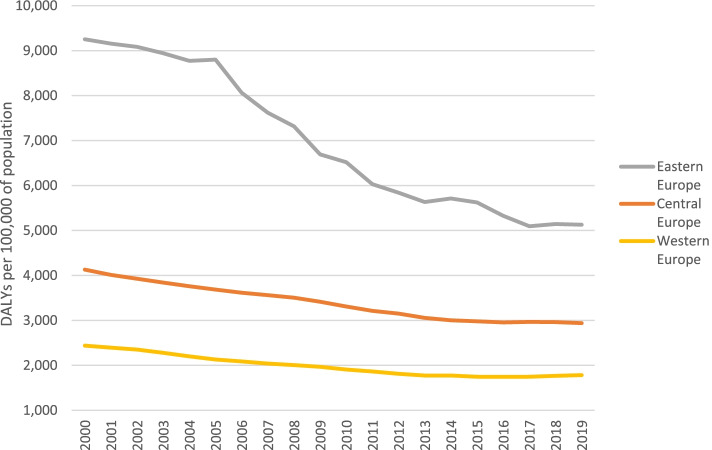
Age-standardized injury DALY rates, by European sub-region, 2000 – 2019

Between 2000 and 2019 injury DALY rates in Eastern, Central and Western Europe have declined by 45%, 29%, and 27%, respectively (see Table 2 and Fig. 3). In Eastern Europe the DALY rates of all cause-of-injury categories declined, with largest declines for conflict and terrorism (-90%), exposure to forces of nature (-90%), and drowning (-62%). In Central Europe the DALY rates of all cause-of-injury categories declined expect for exposure to forces of nature (+ 39%). The largest decreases in Central European injury DALY rates were observed for conflict and terrorism (-76%), drowning (-52%), and poisonings (-49%). In Western Europe largest declines were observed for exposure to forces of nature (-84%), road injuries (-56%), and conflict and terrorism (-54%), whereas increases were observed for police conflict and executions (+ 0.1%), falls (+ 1%), and exposure to environmental heat and cold (+ 46%).

### Inequalities in DALY rates between European countries

**Fig. 4:**
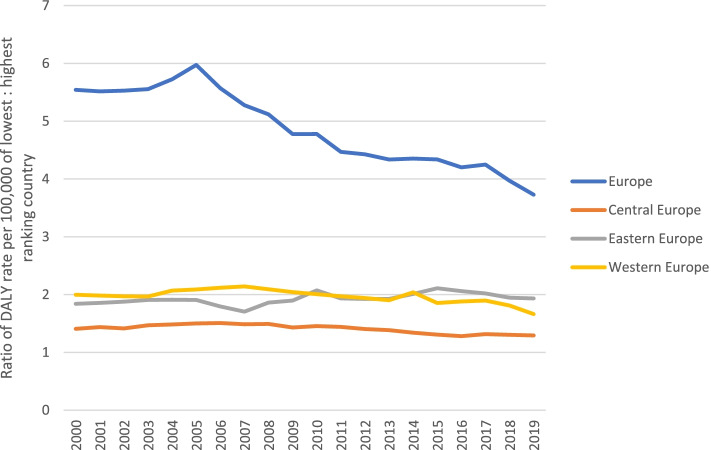
Ratio of DALY rate per 100,000 in the highest to lowest ranking country, for all countries in Europe (Europe) and for European sub-regions (Central, Eastern, Western) between 2000 and 2019

Figure 4 shows the ratio of the DALY rate per 100,000 for highest-ranked to lowest-ranked country in each year from 2000 to 2019. For all European countries, the DALY rate ratio was highest in 2005, with the DALY rate in the lowest-ranking country (Russian Federation) 6.0 times higher compared to the highest-ranking country (Malta). After 2005, the DALY rate ratio between the lowest- and highest-ranking country gradually decreased to 3.7 in 2019. When comparing the injury DALY rates of the lowest- and highest-ranking countries within sub-region over time, we observed that the DALY rate ratio between the lowest- and highest-ranking country in Central Europe, Eastern Europe and Western Europe fluctuated between 1.5 and 1.3, 2.1 and 1.8 and 2.1 and 1.7, respectively.

The DALY rate ratio varied widely by major cause-of-injury and over time. Largest differences in injury DALY rates across countries were observed for interpersonal violence, ranging from 30.5 in 2002 to 12.2 in 2019. For self-harm, the DALY rate ratio declined from 15.3 in 2000 to 8.3 in 2019. For road injuries and falls, the decline in DALY rate ratio were much smaller. For road injury the DALY rate ratio ranged from 6.4 in 2005 to 5.4 in 2019, whereas for falls the DALY rate ratio declined gradually from 3.1 in 2000 to 2.4 in 2019.

## Discussion

### Main findings

In this systematic analysis, we found that over the period from 2000 to 2019, there was an overall reduction in the age-standardized incidence rates due to all injury categories across the European sub-regions. However, slower reductions in injury incidence rates were observed in Central and Western Europe.

Furthermore, we found that mortality and DALY rates of injury varied widely by European sub-region, country, sex and cause-of-injury category. Overall, the injury mortality rate in 2019 in Eastern Europe was twice as high compared to Central Europe and almost three times as high compared to Western Europe. The injury DALY rates showed less pronounced differences between Eastern, Central and Western Europe, although also a distinct East to West gradient was observed. Comparison of injury DALY rates by country showed a fourfold difference between the lowest- and highest-ranking country; however, the difference in injury DALY rates by country was larger for males compared to females. The difference in injury DALY rates between highest- and lowest-ranking country declined from 2005 onwards, indicating declining inequalities in injuries between European countries.

### Comparison to other studies – change over time

From 2000 to 2019 we observed large declines in injury DALY across all European sub-regions; however, largest declines were observed for Eastern Europe. This is comparable to findings from the study by Sethi et al. on injury inequalities in Europe [17]. Particularly in the period 2005 to 2013 the difference in declined injury DALY rates between the Eastern, Central and Western Europe is striking, with rapid progress in Eastern Europe, intermediate progress in Central Europe and slow progress in Western Europe. Several factors may have contributed to the slow progress in Western Europe, including ageing of the population and the fact that Western Europe had much lower DALY rates at the beginning of the period, thus their margin for improvement is much more reduced. However, there are striking differences in all-cause injury DALY rates and injury DALY rates by cause-of-injury categories (e.g., falls and road injuries) across Western European countries. Therefore, it may be worthwhile to assess which injury-specific prevention measures have been taken in Western European countries that showed continuous low or decreasing incidence, mortality, and DALY rates despite ageing of the population. This may lead to the identification of opportunities to reduce the injury DALY rates in Western Europe even further and that may be transferrable to other European countries.

Furthermore, previous studies reported that the financial crisis that hit Europe in 2008 resulted in higher mortality rates, including higher suicide rates [18, 19]. However, for none of the three European sub-regions we observed increasing injury mortality and DALY rates between 2008 and 2011. This finding is broadly in line with earlier results from a systematic analysis on suicide mortality trends among global, national, and regional geographies [20]. The different policy responses and particular characteristics of the societal organizations may help to explain the apparent resilience of the populations to the potentially fatal health effect of an economic downturn.

Despite the large decline in DALY rates resulting from conflict and terrorism, we observed that over this 20-year study period the burden of terrorism remained at its peak in Croatia, Serbia, and Bosnia and Herzegovina. An explanation for this may be that the Bosnian War of the early 1990s had a profound impact on health and disabilities, and that many Balkan inhabitants may therefore still be experiencing the long-term consequences of injury, almost 30-years later [21].

In addition, from 2000 to 2019, Eastern Europe had the highest injury mortality rates attributable to cold or hot temperatures. This may be in part explained by the fact that in Eastern Europe the 2003 and 2010 heat-waves led to an increased number of deaths [22, 23]. Moreover, a study evaluating the global, regional, and national mortality burden associated with non-optimal ambient temperatures showed that between 2000 and 2019, Eastern Europe had the highest heat-related excess mortality and that this rate was two to five times higher compared to other European regions in the same period [24]. Climate change is expected to affect populations’ health by increasing the mortality burden [25]; national prevention plans are therefore needed to reduce the heat- and/or cold-related impact on the injured.

### Comparison to other studies – inequalities in injury

Our findings suggest that health inequalities associated with injuries between European countries decline over time. This is in contrast to the findings of two cross-country studies that reported increasing inequalities across Europe over time [15, 16]. Reasons for these differences in findings may be the different metrics that were used to measure health inequalities, namely mortality ratios versus DALY rate ratios. Second, there are differences in the populations that were studied. Göpfert et al. [15] and Sethi et al. [16] studied age mortality rates among children aged 0 to 14 years old in 53 countries included in the WHO European region, whereas our study included all ages in 42 European countries. Third, there were differences in the period that was studied. Göpfert et al. [15] and Sethi et al. [16] reported differences in injury mortality rates for the years 2000 and 2011 and 2015, respectively to measure differences in health inequalities over time, whereas in our study differences in DALY rate ratios from 2000 to 2019 were reported.

From 2003 to 2005 the observed inequalities in DALY rate ratio increased. Main reason for this was that the DALY rate in the Eastern European region increased during this period. An explanation for this finding may be the impact of dissolution of the former Soviet Union and its social and economic consequences on health and mortality in subsequent years [26]. However, others have argued that causes of the increased mortality rates in Eastern European countries are more intricate and may be the result of a combination of lifestyle habits, economic impoverishment, widening social inequality and the breakdown of political institutions [27, 28]. From 2005 onwards, DALY rates in Eastern Europe have decreased more rapidly compared to Central and Western Europe. A possible contributing factor may be the anti-alcohol policies implemented in Russia in 2005–2006, although other factors, such as economic growth and national initiatives to combat the road safety, childhood injury prevention efforts and violence prevention most probably have played a role as well [29–31]. Therefore, governments should consider to introduce stricter preventive policies including marketing controls and/or use of taxation to reduce the injury disease burden across European countries.

### Strengths and limitations

A strength of this systematic analysis is that the DALY metric was used to assess the population health impact of injuries in Europe, describe trends over time and inequalities in injuries across countries. The DALY incorporates mortality and disability, which allows for a more complete assessment of the population health impact. Previous studies that investigated injury inequalities across European countries were based on mortality rate ratios [15, 16] rather than DALY rate ratios.

A second strength of this study is that the mortality rate estimates in European countries were based on complete cause-of-death registration systems [32]. However, a limitation is that nationally representative injury incidence data – essential input for the YLD calculations – were available for 19 of the 44 included countries, of which many datasets were collected 10 or more years ago. Incidence estimates for every European country and recent years were made by using statistical models that use available data on incidence, prevalence, remission, duration and extra risk of mortality due to the injury from the year and country for which incidence is estimated, as well as from previous years and other countries, but these estimates are inherently less accurate for countries without national representative incidence data [1–3].

A second limitation is that the cause versus nature-of-injury matrices, required for the injury YLD calculations, were based on outpatient, inpatient, and emergency room discharge data from an even smaller number of countries, namely seventeen European countries that are spread across the three European regions (Bulgaria, Cyprus, Czechia, Denmark, Estonia, Hungary, Iceland, Italy, Latvia, North Macedonia, Malta, Netherlands, Norway, Portugal, Slovenia, Spain, and Sweden).

A third limitation of our study is that the analytical approach chosen to explore inequalities associated to injuries across countries focuses on the extremes by calculating rate ratios between countries with highest and lowest injury rates. The GBD study does not provide DALY rates for sub-groups of the population, by socio-economic status or on a small area deprivation level [2]. As a result, we were not able to investigate health inequalities within countries over time. Therefore, we did not investigate injury inequalities by age groups and sex.

Finally, another limitation of our study is that the DALY estimates were based on prevalence-based data. The epidemiological Disease Modeling – Metaregression (DisMod-MR) software tool is used to stream out prevalence from incidence, and this process assumes a steady state where rates are not changing over time [1]. This steady-state assumption may lead to inaccurate estimates of prevalence of long-term disability if there are large trends in incidence rates or mortality.

## Conclusions

Injuries in Europe are still a major public health problem. In 2019 across all European region countries, 109.7 million people sustained injuries that warranted some type of healthcare and 458,669 people died from injuries. However, mortality and DALY rates of injury varied widely by European region, country, sex and cause-of-injury category. Injury mortality and DALY rates were highest in Eastern Europe and lowest in Western Europe, although differences in injury DALY rates declined rapidly, particularly in the past decade. The injury DALY rate ratio of highest- and lowest-ranking country declined from 2005 onwards, indicating continuous declining inequalities in injuries between European countries.

## Data Availability

Data are available in a public, open access repository (ghdx.healthdata.org). The data that support the findings of this study are available from the corresponding author upon reasonable request.

## Abbreviations

DALY: Disability-Adjusted Life Year
GBD: Global Burden of Disease
ICD: International Classification of Diseases
YLD: Years Lived with Disability
YLL: Years of Life Lost
